# Response to “high-flow nasal cannula and intubation risk in severe PjP: methodological and clinical perspectives”

**DOI:** 10.1186/s13613-025-01573-6

**Published:** 2025-11-17

**Authors:** Florian Reizine, Vicky Stiegler, Benoit Tessoulin, Nahema Issa, Benjamin Gaborit

**Affiliations:** 1 Service de réanimation polyvalente, Centre Hospitalier de Vannes, 56000 Vannes, France; 2 Department of Infectious Diseases, University Hospital of Nantes and Centre d’Investigation Clinique 1413, INSERM, 1 Place Alexis-Ricordeau, 44000 Nantes, France; 3 INSERM, U1232, Hematology Department, Nantes University Hospital, CRCI2NA, Nantes University, Nantes, France; 4 Internal medicine and Infectious Disease Unit, Groupe Saint-André, University Hospital, Bordeaux, France; 5 EA 3826, Laboratory of clinical and experimental therapeutics of infections, IRS2-Nantes Biotech, Nantes, France

**Keywords:** Pneumocystis jirovecii pneumonia, acute hypoxemic failure, HFNC, NIV

To the Editor,

We thank Wang and Cheng for their thoughtful comments [1] on our study of respiratory management in critically ill patients with *Pneumocystis jirovecii* pneumonia (PjP) [2]. We welcome the opportunity to clarify several methodological points.

## Competing-risk analysis with center and epoch adjustment

We agree that censoring patients who died without intubation may overestimate the “intubation-free” probability. In response, we performed an additional Fine–Gray competing-risk analysis, treating death prior to intubation as a competing event. We also adjusted for variability across centers and temporal changes in practice (2011–2014, 2015–2018, 2019–2021). In this model, high-flow nasal cannula (HFNC) remained independently associated with a reduced subdistribution hazard of intubation compared with standard oxygen (sHR = 0.46; 95% CI 0.28–0.78; p = 0.004), while non-invasive ventilation (NIV) showed no significant effect (sHR = 0.84; 95% CI 0.51–1.36; p = 0.47). These findings confirm the robustness of our primary results after accounting for temporal and inter-center heterogeneity.

## Classification of alternating HFNC/NIV patients

Patients who alternated between HFNC and NIV primarily received repeated NIV sessions, with HFNC used for comfort between sessions. We therefore classified them under “NIV,” which reflects bedside practice more accurately than a multi-state model that would overemphasize short HFNC intervals. Only a randomized trial could definitively disentangle the respective contributions of HFNC and NIV.

## Residual confounding and scope of additional analyses

We acknowledge that residual confounding remains possible despite inverse probability weighting and adjustment for center and epoch. A fully multivariable Fine-Gray model including all baseline covariates would require a dedicated analysis and exceeds the scope of this letter. Our goal was to verify that the association between HFNC and lower intubation risk persisted under competing-risk modeling and key sources of heterogeneity, an objective that these additional analyses achieves.

## Subgroups, prophylaxis, and standardized protocols

As noted, poor prognosis in patients with long-term corticosteroid use, solid tumors, or high SOFA scores is an important issue. Exploratory subgroup analyses did not reveal significant interactions with HFNC, but our sample size was insufficient for firm conclusions. Reinforcing pneumocystis prophylaxis and implementing standardized HFNC escalation strategies (e.g., ROX index thresholds, early awake prone positioning, predefined intubation criteria) are promising avenues for improving patient outcomes and should be evaluated prospectively.

## Conclusion

Our study provides real-world evidence that HFNC is associated with a lower risk of intubation in severe PjP, although no survival benefit at day 90 was observed. The additional competing-risk analysis supports the robustness of this finding, while underlining the inherent limitations of retrospective data. Ultimately, only a prospective randomized controlled trial will definitively establish the role of HFNC versus alternative oxygenation strategies in critically ill PjP patients.

## Data Availability

Not applicable.
